# Role of noncoding RNAs in cardiac ageing

**DOI:** 10.3389/fcvm.2023.1142575

**Published:** 2023-03-22

**Authors:** Lijo N. Varghese, Daryl O. Schwenke, Rajesh Katare

**Affiliations:** Department of Physiology, HeartOtago, School of Biomedical Sciences, University of Otago, Dunedin, New Zealand

**Keywords:** cardiac ageing, cardiovascular disease, molecular changes, non-coding RNA, microRNA, long non-coding RNA

## Abstract

The global population is estimated to reach 9.8 billion by 2050, of which 2.1 billion will comprise individuals above 60 years of age. As the number of elderly is estimated to double from 2017, it is a victory of the modern healthcare system but also worrisome as ageing, and the onset of chronic disease are correlated. Among other chronic conditions, cardiovascular diseases (CVDs) are the leading cause of death in the aged population. While the underlying cause of the age-associated development of CVDs is not fully understood, studies indicate the role of non-coding RNAs such as microRNAs (miRNAs) and long noncoding RNAs (lnc-RNAs) in the development of age-associated CVDs. miRNAs and lnc-RNAs are non-coding RNAs which control gene expression at the post-transcriptional level. The expression of specific miRNAs and lnc-RNAs are reportedly dysregulated with age, leading to cardiovascular system changes and ultimately causing CVDs. Since miRNAs and lnc-RNAs play several vital roles in maintaining the normal functioning of the cardiovascular system, they are also being explored for their therapeutic potential as a treatment for CVDs. This review will first explore the pathophysiological changes associated with ageing. Next, we will review the known mechanisms underlying the development of CVD in ageing with a specific focus on miRNA and lnc-RNAs. Finally, we will discuss the therapeutic options and future challenges towards healthy cardiac ageing. With the global ageing population on the rise, this review will provide a fundamental understanding of some of the underlying molecular mechanisms of cardiac ageing.

## Introduction

Ageing is a natural, ineludible, and universal process for most species (1). Biological ageing is often characterised as the gradual decline in the normal physiological functioning of tissues and organs (2). Advancements in medical technology, improved sanitation facilities, availability of a nutritious diet, and better healthcare facilities have increased the global average life expectancy from 66.8 years in 2000 to 73.4 years in 2019, which is the fastest since 1950 (3, 4). This spurt in life expectancy is estimated to double the population ≥60 years of age, from 1 billion in 2017 to 2.1 billion in 2050 (5). However, increased life span is correlated with the prevalence of chronic illnesses such as cardiovascular disorders (CVDs), hypertension, diabetes, cancer, and neurodegenerative disorders (6). Among these, CVDs rank as the leading cause of death globally, with 18.6 million deaths yearly (7).

**Table 1:** Non-coding RNAs associated with cardiac ageing.

Non-coding RNA	Modulation in ageing	Molecular target	Effect	Organism	Reference
***MicroRNA***					
*miR-34a*	Upregulated	PNUTS SIRT1	Cardiomyocyte apoptosis	Mice Human	(88, 112)
*miR-17-3p*	Downregulated	PAR-4	Fibroblast apoptosis	Mice cardiac fibroblast cell line	(120–122)
*miR-18a*	Downregulated	TSP-1	Cardiac fibrosis	Mice	(123)
*miR-19a/b*	Downregulated	CTGF	Cardiac fibrosis	Mice	(123)
*miR-21*	Upregulated	SPRY1 PTEN	Cardiac fibrosis	Mice	(124, 128)
*miR-22*	Upregulated	Mimecan	Cardiac fibrosis	Mice	(134)
*miR-29*	Upregulated	ECM proteins	Reduces cardiac fibrosis	*Nothobranchius furzeri*	(141)
***Long non-coding RNA***					
*MALAT1*	Downregulated	miR-34a	Cardiomyocyte apoptosis	Rat	(158, 159)
*SARRAH*	Downregulated	Caspase	Cardiomyocyte apoptosis	Mice	(161, 162)
*CHRF*	Upregulated	Myd88, miR-93	Cardiac hypertrophy	Mice	(46, 164)
*MIRT*	Upregulated	TGFβ, STAT3, ICAM1	Left ventricular remodeling	Mice	(165)

Non-coding RNAs associated with cardiac ageing: PNUTS, Phosphatase-1 nuclear-targeting subunit; SIRT1, Sirtuin 1; PAR-4, Protein prostate apoptosis response-4, TSP-1, Thrombospondin- 1; CTGF, Connective tissue growth factor; SPRY1, Sprout protein homolog 1; PTEN, Phosphatase and tensin homolog; ECM proteins, Extracellular matrix proteins (collagen, fibrillin, and elastin), MYD88, Myeloid differentiation protein-88; TGFβ, Tissue growth factor β; STAT3, Signal transducer and activator of transcription 3; ICAM1, Intercellular adhesion molecule 1.

CVDs were earlier thought to be rampant in developed countries. However, that trend has dramatically changed due to modernisation. The numbers are steadily increasing in developing countries such as India and China, which account for most of the global population (7, 8) Among the several risk factors for CVDs, such as an unhealthy diet, lack of physical activity or exercise, smoking, and alcohol abuse, cardiac ageing is the cardinal factor in the development of CVDs (9, 10). More than 50% of individuals above 65 years of age suffer from some form of cardiac disorder. With a steady increase in the aged population, there is tremendous pressure on the healthcare system to aid the number of people affected by CVDs (11). A deeper understanding of the physiological and pathological processes in the cardiovascular system with ageing will help develop novel measures for healthy cardiac ageing and thereby reduce the burden of CVD-related deaths (12).

## Pathophysiological changes associated with cardiac ageing

The cardiovascular system undergoes several pathophysiological alterations with age, which become more notable as the heart's reparative mechanisms gradually decline (13). These changes in the aged heart can differ between individuals and thus differ from the chronological age of the individual (14). Myocardial remodeling, microangiopathy, diastolic dysfunction, arrhythmia, and heart failure are some alterations that occur with cardiac ageing (11, 15) Loss of cardiomyocytes with age increases the risk for the development of cardiomyopathies, ischemic heart disease, and heart failure (16). These dead cardiomyocytes are often replaced with fibrous tissue (17). Accumulation of fibrous tissue, irrespective of cardiomyocyte apoptosis, is a hallmark of cardiac ageing which eventually impairs cardiac contractility, particularly diastolic function, commonly referred to as heart failure with preserved ejection fraction (18).

Ageing has a prominent effect not just on the cardiomyocytes but also on the pacemaker cells and valves of the heart. A gradual decrease in the number of pacemaker cells in the sinoatrial node is observed with age (19). The loss of pacemaker cells leads to a progressive decrease in the intrinsic heart rate, reducing the blood supply to vital organs (20, 21). In addition, ageing-induced thickening and calcification of the aortic valve cusps lead to aortic sclerosis, which is observed in around 20% of individuals above 65 years. Aortic sclerosis can develop into a more serious aortic valve stenosis (22). Aortic valve stenosis reduces the outflow of blood from the left ventricle, resulting in left ventricular (LV) hypertrophy as a compensatory mechanism to overcome pressure overload (23). Like the aortic valve, the mitral valve, which guards blood flow against the left atrium to the left ventricle, also shows increased deposition of collagen, lipids, and calcium with ageing. Excess deposition of calcium causes mitral annular calcification, which causes an increased risk of stroke, endocarditis, and necrosis of the calcified tissue (22).

In addition to the changes in the cardiac cells, studies have also reported impairment in vascular structure and function (24). Thickening and stiffening of the arteries are prominent features of ageing caused by increased deposition of collagen and reduced elastin. Arterial stiffness can lead to myocardial infarction (MI) due to a sustained increase in systolic pressure (15). In addition, ageing causes endothelial dysfunction in blood vessels, which can be attributed to increased inflammation and oxidative stress (25). Endothelial dysfunction is one of the first steps in the progression of atherosclerosis (26). While the exact mechanism of these ageing-induced changes is yet to be explored, they can occur due to the ageing-induced molecular changes in the cardiac cells.

## Ageing-induced molecular changes in cardiovascular cells

**Figure 1:**
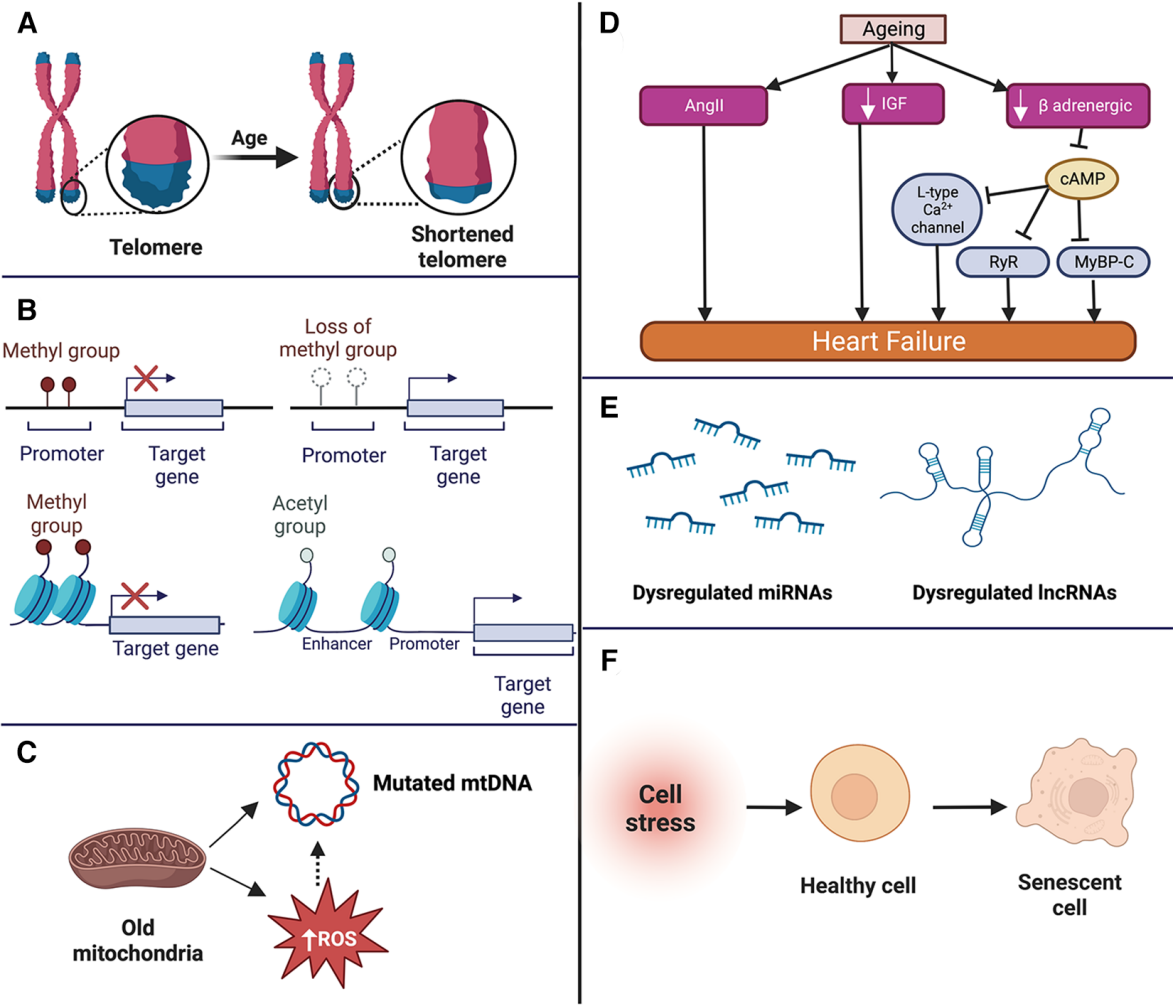
Summary of the molecular changes that occur in the aged heart such as telomere shortening (**A**), epigenetic changes (**B**), mitochondrial dysfunction (**C**), neurohormonal changes (**D**) dysregulation of non-coding RNAs (**E**), and cellular senescence (**F**). mtDNA, mitochondrial DNA; ROS, reactive oxygen species; miRNA, microRNA; lncRNA, long non-coding RNA; AngII, angiotensin II; IGF, insulin like growth factor; RyR, ryanodine receptor; MyBP-C, f Apoptosis of cardiomyocytes, loss of pacemaker cells, thickening and calcification of the valves, and impairment of vascular structure and function.

Ageing-induced molecular changes in the cardiac cells form the basis of the pathophysiological changes observed in aged individuals (27). Among all the molecular changes, telomere shortening, epigenetic changes, mitochondrial dysfunction, and impaired intercellular communication are the hallmark changes observed in cardiac ageing (28, 29) (summarised in Figure 1).

### Telomere shortening

Telomeres are looped DNA (TTAGGG) repeats at the ends of the chromosomes, protecting the chromosome from degradation (30). The length of telomeres reduces progressively with each cell cycle, primarily due to the inability of DNA polymerase to completely replicate the telomeres (31). When the telomeres shorten to a critical length, the cell cycle is arrested, and the cell enters a state of senescence and activates apoptosis (32). The length of the telomere is maintained by an enzyme called telomerase (31). Telomerase enzyme contains two components: Telomerase RNA component (TERC) and telomerase reverse transcriptase (TERT). TERC includes a template for telomeric DNA, which is used by TERT to synthesise new telomeric DNA repeats. Telomerase activity reduces with age (33), leading to short telomeres, and short telomeres have been linked to the progression of cancer, diabetes, and CVDs (34). In a study conducted on *TERC*^−^/^−^ mice, the catalytic unit of the telomerase enzyme, mice displayed thinning of ventricular walls and cardiomyocyte apoptosis (35). Brouillette et al. conducted a trial with 1,500 participants to investigate the correlation between telomere length and the occurrence of coronary heart disease. They observed that the mean telomere length reduced by around 9% each year and that the participants with short telomere length had a higher risk of developing coronary heart disease than those with comparatively longer telomeres (36). Similarly, coronary endothelial cells with short telomeres exhibit impaired function and formation of plaques leading to age-dependent coronary artery disease (37, 38). Despite several studies indicating the role of short telomeres in the progression of CVDs, a large study including 7,827 participants found telomere length was not significantly associated with cardiovascular mortality (39). The reason for the inconsistency can be multi-factorial such as ethnicity of individuals, medical history, and other factors such as hypertension, smoking, and obesity, as these can attribute to telomere shortening (39, 40). Thus, using telomere length as a marker for age-associated CVD needs more conclusive evidence (41).

### Epigenetic changes

Epigenetic changes, including methylation of DNA, RNA methylation, and histone modifications, have been demonstrated to be associated with the development of ageing-induced CVDs (42). DNA methylation involving methylation at 5-cytosine is a highly conserved process in plants, animals, and fungi (43). DNA methylation and demethylation are essential for regulating gene expression, splicing, transposon silencing, and genomic instability (44). DNA methylation at the promoter regions inhibits the binding of transcription factors, which suppresses transcription, whereas demethylation leads to gene expression (45). Studies have identified DNA hypermethylation with age (increased DNA methylation) (44, 46). DNA hypermethylation has been observed in atherosclerotic lesions, as hypermethylation represses the expression of atheroprotective genes such as kruppel like factor 4 (*KLF4*) and ATP binding cassette subfamily A member 1 (*ABCA1)* (44)*.* Like DNA methylation, RNA is also methylated. The most abundant modification is the methylation of adenosine in mRNA (N6-methyladenosine, M6A), which is catalysed by the enzyme methyltransferase-like 3 (METTL3) (47). N6-methyladenosine is also found in other RNAs, such as transfer RNA, ribosomal RNAs, and other non-coding RNAs (48). Even though RNA methylation profile changes with age are not elucidated, it has been linked to cardiac hypertrophy, which is frequently observed with ageing (48). Dorn et al. demonstrated cardiac hypertrophy in METTL3 overexpressing mice by 8 months of age. The mechanism by which METTL3 overexpression causes hypertrophy is still being explored. However, initial findings indicate it increases the expression of the β-myosin heavy chain (β-MHC), a critical hypertrophic protein. In vitro analysis of METTL3 inhibition attenuates cardiomyocyte hypertrophy, indicating a role in the development of cardiac hypertrophy (48).

Histones are proteins which wrap DNA to form the nucleosome. The epigenetic modifications of histones, such as methylation, acetylation, carbonylation, and phosphorylation, are associated with CVDs (49). Like DNA methylation, histone modifications repress transcription (47). Histone methylation is mediated by histone methyltransferases and demethylases, which add and remove methyl groups from histones, respectively (47). Changes in the histone methylation profile have been observed in cardiac hypertrophy, atherosclerosis, and dilated cardiomyopathy. SET and MYND domain-containing protein 1 (SMYD1) is a histone methyltransferase which represses the activity of pro-hypertrophic genes such as transforming growth factor-β-3 (TGFβ3) and natriuretic peptide A (NPPA) and its depletion is associated with hypertrophy (49). However, there is no conclusive evidence that the histone methylation pattern changes with age. Histone acetyltransferases add an acetyl group from acetyl-CoA to lysine residues, and deacetylases remove the acetyl group (47). Histone deacetylase (HDAC) is catalysed primarily by HDAC1 and HDAC2, and their global deletion has been linked to cardiac arrhythmia and dilated cardiomyopathy due to the abnormal growth of cardiomyocytes (50). Whether changes in histone acetylation lead to age-associated CVDs, is not fully understood and is an area of active research (47).

### Mitochondrial dysfunction

Mitochondria have a double membrane consisting of inner and outer layers. The inner layer comprises 4 complexes—Complex I to IV, which generate ATP *via* oxidative phosphorylation (51). With age, oxidative phosphorylation declines primarily because of the dysfunction of complexes I and IV, reducing the production of ATP in the cells (52). This dysfunction can be due to the accumulation of damage to the mitochondrial DNA (mtDNA) and excess amounts of reactive oxygen species (ROS) (53). Increased ROS accelerates atherogenesis by oxidising low-density lipoprotein, promoting endothelial cell senescence and apoptosis (54). Oxidised low-density lipoproteins increase the risk of plaque formation and subsequent atherosclerosis by reducing the availability of nitric oxide and promoting inflammation, leukocyte adhesion, and smooth muscle cell proliferation (55). Increased ROS-linked endothelial cell apoptosis also impairs blood flow to the heart and the brain due to rarefaction of the blood vessels (56). ROS is also associated with cardiomyocyte necrosis and the progression of cardiac hypertrophy (57, 58). ROS generated by monoamine oxidase (MAO), an enzyme present on the outer membrane of the mitochondria, induces cardiomyocyte necrosis by activating p53 (10, 58). ROS also induces cardiac hypertrophy through the activation of pro-hypertrophic signalling kinases and transcription factors such as mitogen-activated protein kinase (MAP-kinase) and nuclear factor kappa light chain enhancer of activated B cells (NF-*κ*B) (59, 60). Thus, increased ROS generation due to age-dependent mitochondria dysfunction is a crucial molecular change associated with cardiac ageing and the development of CVDs (59).

Mitochondria is a unique cell organelle, as it has its DNA known as mitochondrial DNA (mtDNA). mtDNA is circular and has genes that encode proteins necessary to produce energy (61). The mtDNA accumulates mutations with age, which can also be caused due to age-impaired production of excess ROS (62). mtDNA mutations have been linked with several diseases, such as Alzheimer's disease, Parkinson's disease, CVDs, diabetes, and cancer (63). Lindroos et al. studied the effect of one of the mtDNA mutations (m.3243A > G) in 14 individuals. This mutation affects the translation of respiratory chain complex proteins by impairing the tRNA necessary for the translation (64, 65). The study revealed impaired glucose oxidation and lower stroke volume in individuals with mutation (65). Studies have also identified an association of other mtDNA mutations, such as m.3256C > T, m.12315G > A, m.13513G > A, and m.15059G > A with atherosclerosis due to impaired translation of proteins essential for oxidative phosphorylation and increased production of ROS (66). While studies suggest a strong correlation between mtDNA, ageing, and CVD, whether mtDNA is the cause of CVD remains unclear, warranting future studies to ascertain the mechanisms and its reliability as an age-associated marker of CVDs (63).

### Signalling pathways

Ageing has a detrimental effect on the neuro-hormonal signalling pathways essential for the functioning of the cardiovascular system. One of the signalling pathways linked to the age-dependent progression of cardiac function decline and CVD is the renin-angiotensin-aldosterone system (RAAS) (59). RAAS regulates blood pressure by controlling fluid and electrolytes through a well-coordinated balance between the liver, kidneys, heart, lungs and blood vessels (67). In brief, RAAS is activated in the kidneys by a drop in blood pressure, leading to renin's activation. Active renin is secreted in the blood, which converts inactive angiotensinogen (produced by the liver) to angiotensin I. Angiotensin I is converted by an angiotensin-converting enzyme (primarily present in the lungs) to angiotensin II (Ang II). Ang II is the central effector molecule of the RAAS system, which acts on the adrenal gland, brain, and blood vessels to elevate blood pressure by various mechanisms (68). Ageing increases Ang II concentration, which is associated with cardiac hypertrophy, fibrosis, and increased ROS (68). Another significant signalling pathway affected by age is the insulin-like growth factor-1 (IGF), which reduces with age in humans. While the reduction in the IGF signalling pathways has been observed to improve cardiac function with age in *Drosophila* and mice models, the same has not been observed in humans (59). Vasan et al. examined the levels of IGF-1 in 717 elderly individuals with no known history of myocardial infarction and congestive heart failure for a mean period of 5.2 years. Of the 717 elderlies, 56 developed congestive heart failure (69).

Interestingly, all these individuals had serum IGF-1 levels below the median value—the exact reason for these contradictory findings between different species of ageing warrants further studies (59). Beta-adrenergic signalling is an important signalling pathway which regulates heart rate and contractility. There are 3 major beta-adrenergic receptors—β1, β2, and β3 receptors, which are G-coupled protein receptors. In brief, ligands (major catecholamines such as epinephrine and norepinephrine) bind to the beta receptors, which activate adenylyl cyclase and increase the level of cyclic adenosine monophosphate (cAMP). cAMP targets protein kinase A, which activates several proteins such as ryanodine receptors, L-type calcium channels and myosin binding protein-C, eventually leading to cardiomyocyte contraction (70). With age, the beta-adrenergic signalling reduces, primarily due to impaired agonist binding to the receptor and reduced β1 receptors (70, 71). A decreased beta-adrenergic response seems beneficial, especially in the aged heart, as it reduces the risk of arrhythmia, hypertrophy, and apoptosis (70, 72). However, the lower beta-adrenergic response is also associated with lowered exercise tolerance, impaired autonomic regulation and impaired arterial-ventricular load (70, 72, 73).

### Cellular senescence

Cellular senescence is a complex, dynamic, and multi-step process in which a cell undergoes permanent cell cycle arrest with continued metabolic activities (74). Cellular senescence is induced as a response to stressors such as DNA damage, telomere shortening, oxidative stress, mitochondrial damage, which are a result of ageing (74, 75). A senescent cell has multiple characteristic features such as an enlarged and flattened morphology, senescence associated beta-galactosidase activity, activation of senescence associated secretory phenotype (SASP), and DNA damage response (74, 76). SASP is a prominent hallmark of a senescent cell, as it releases pro-inflammatory chemokines and cytokines, growth factors, ECM proteins, and activation of p16 and p21, which affects the neighboring cells or distant cells, if they are released in the systemic circulation (76). SASP has a deleterious effect, as it can induce senescence in the neighboring healthy cells and even 10%–15% of senescent cells in a tissue, can cause tissue degeneration (77, 78). Cellular senescence has been linked to age-related diseases such as osteoporosis, renal diseases, neurodegenerative diseases, pulmonary fibrosis, and CVDs (79).

The heart is a mosaic of different cell types such as cardiomyocytes, vascular smooth muscle cells, endothelial cells, and fibroblasts, and senescence of individual cell types can cascade to a disease (80). For example, senescent cardiomyocytes along with the phenotypical changes of a senescent cell exhibit contractile dysfunction and hypertrophic growth, which eventually leads to cardiac hypertrophy, arrhythmias, cardiac remodeling, and heart failure (81). Senescent endothelial cells have impaired production of endothelin-1 (vasoconstrictor) and nitric oxide (vasodilator) which affects vascular function. Senescent endothelial cells have been studied to develop disorders such as atherosclerosis and heart failure with preserved ejection fraction (80). Senescent fibroblasts secrete IGF-1 which promotes collagen synthesis and exacerbates cardiac fibrosis. It also induces senescence in neighboring cardiomyocytes through paracrine signalling (80). Similarly, senescent vascular smooth muscles lead to atherosclerosis and pulmonary hypertension (82).

Senolytics are a class of drugs which selectively induces apoptosis in senescent cells by activating the B-cell lymphoma 2 (BCL-2) family proteins, p53, phosphoinositide-3-kinases (PI3K) and other apoptotic pathways (83). Salerno et al. combined Dasatinib and Quercetin, two senolytic drugs and administered to 22–24 months old mice after acute MI, released healthy cardiac stem cells and improved cardiac remodeling and regeneration, which eventually improved LV function. Thus, removal of senescent cells by senolytics improves the overall functioning of the heart, even in aged mice (77, 83). In a recent study Cattaneo et al. reported the importance of longevity-associated BPIFB4 (*LAV-BPIFB4*) gene in supporting cardiac function and vascularization in ageing cardiomyopathy (84). They demonstrated reduced *LAV-BPIFB4* in older hearts and that gene therapy with *LAV-BPIFB4* rescued cardiac function and myocardial perfusion in aged mice by improving microvasculature density and pericyte coverage. Therefore, therapeutic modalities targeting cellular senecesnce may have therapeutic potential although long-term studies required to determine the sustainability of the effect.

### Non-coding RNAs

The human genome consists of only 3% protein-coding genes, whereas most of the genome is transcribed to produce non-coding RNAs (nc-RNAs) (85). These nc-RNAs include a wide array of molecules such as microRNAs (miRNAs), long-noncoding RNAs (lncRNAs), small nuclear RNAs, small nucleolar RNAs, piwi-interacting RNA, circular RNA and transfer RNA etc (86).. Based on their function, these nc-RNAs can be classified into two categories: housekeeping RNAs and regulatory RNAs. Housekeeping RNAs, such as ribosomal RNA, transfer RNA, and small nuclear RNA, are present in virtually all cells and are essential for the cell's normal functioning. Regulatory RNAs such as piwi-RNA, miRNAs, and lncRNAs regulate gene expression at the transcriptional or translational levels (86, 87). Among the ncRNAs, miRNAs and lncRNAs are gaining prominence in ageing-associated diseases. The expression of miRNAs and lncRNAs dysregulate with age which leads to the onset and progression of ageing-associated diseases such as CVDs (88, 89), Alzheimer's (90), cancer (91), and diabetes (92). Furthermore, miRNAs such as miR-146a/b, -126, -34a, -22 are associated with inducing senescence in vascular smooth muscle cells, endothelial cells, cardiomyocytes, and fibroblasts, respectively (80, 93). The role of lncRNAs with respect to senescence is an active area of research, although to date only few lncRNAs that induce cellular senescence have been identified. For example, senescence associated lncRNA-1 triggers senescence in fibroblasts and lncRNA-H19 triggers senescence in cardiomyocytes (89, 94). Several studies have demonstrated the critical role of miRNAs and lncRNAs in cardiovascular system homeostasis and that their dysregulation with age leads to CVDs (95, 96). In the next section, we will focus on non-coding RNAs (miRNAs and lnc-RNAs) in the age-dependent development of CVDs.

## MicroRNAs and cardiac ageing

MicroRNAs (miRNAs) are nc-RNAs that are ∼22–25 nucleotides long (97). Since their discovery in 1993 in Caenorhabditis elegans, miRNAs have been found in many organisms, including humans. It is estimated that the mammalian genome codes for more than 2000 miRNAs, and around 60% of coding genes are regulated by miRNAs (98). Several years into their discovery, the role and function of miRNAs were not fully understood. However, the discovery that two miRNAs—lin-4 and let-7 control nematode development led to a more thorough study of these small nc-RNAs (99). miRNAs inhibit protein synthesis by translational repression or mRNA degradation (100). Furthermore, each miRNA can control the expression of more than one mRNA. Hence, each mRNA can be a target for more than one miRNA (99). Thus, miRNAs control the gene expression of several proteins required for essential cellular and metabolic pathways that control cell growth, differentiation, and survival (101, 102). Concerning the cardiovascular system, several miRNAs are involved in cardiac development, differentiation, and normal function (102). Deletion or dysregulation in the expression of miRNAs leads to cardiac dysfunction and eventually death, highlighting the crucial role of miRNAs in the functioning of the cardiovascular system (102).

### MicroRNA biogenesis

**Figure 2:**
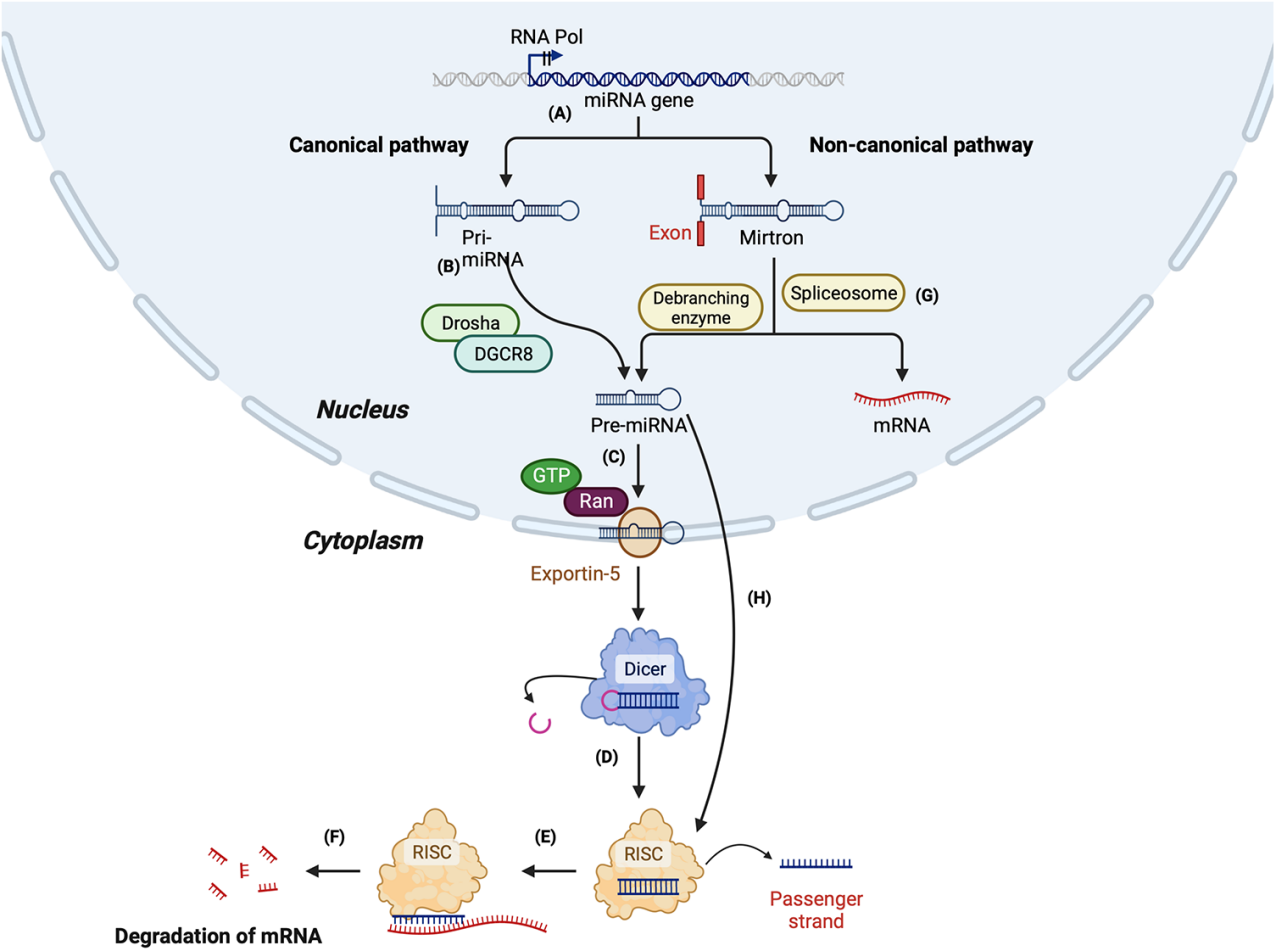
Summary of miRNA biogenesis *via* canonical and non-canonical pathway. Canonical pathway: (**A**) The miRNA gene is transcribed by RNA polymerase II to form a primary mi-RNA (pri-miRNA). (**B**) The pri-miRNA is cleaved by Drosha and Di-George syndrome critical region 8 (DGCR8) to form the precursor miRNA (pre-miRNA). (**C**) Pre-miRNA is transported to the cytoplasm by exportin-5. (**D**) In the cytoplasm, pre-miRNA is cleaved by Dicer to form a duplex mature miRNA. (**E**) The mature miRNA is loaded to RNA-induced silencing complex (RISC), leading to the cleavage of the passenger strand. (**F**) miR-RISC binds to the target mRNA leading to its degradation. Non-canonical pathway: (**G**) Synthesis of miRNA by Drosha/DGCR8 independent pathway: The primary miRNA is spliced by a spliceosome, forming a branched pre-miRNA. The pre-miRNA is debranched by a debranching enzyme, after which the synthesis is similar to the canonical pathway. (**H**) Synthesis of miRNA by Dicer-independent pathway: The pre-miRNA formed by the cleavage of pri-miRNA by Drosha/DGCR8 is exported to the cytoplasm. The pre-miRNA is not long enough to be processed by Dicer and forms the RISC.

The biogenesis of miRNAs begins in the nucleus by the most common canonical or less common non-canonical pathway (summarised in Figure 2). In the canonical pathway, the primary transcript (pri-miRNA) transcribed by RNA-polymerase II is a several hundred nucleotides long transcript, which is processed to form a 70–100 nucleotide long precursor known as pre-miRNA (103, 104). Next, pri-miRNA is cleaved by a protein complex called the microprocessor, which comprises two enzymes—Drosha and DiGeorge syndrome critical region gene 8 (DGCR8) to form a shorter pre-miRNA. DGCR8, an RNA binding protein, binds to the pri-miRNA and Drosha cleaves it at the base of the hairpin structure, which results in a 2 nt 3′ overhang. Next, a nuclear transport receptor protein called exportin-5, along with RAN-GTPase, moves the pre-miRNA from the nucleus to the cytoplasm. Once in the cytoplasm, Dicer, an RNase III endonuclease, removes the terminal loop from the pre-miRNA to form the mature miRNA consisting of 18–22 nucleotides (104, 105). Finally, the mature miRNA duplex is loaded to RNA-induced silencing complex (RISC), a multi-protein complex consisting of Argonaute protein (Ago2). Once loaded on the RISC complex, one of the duplex strands, known as the passenger strand, is released, while the other strand, the guide strand, remains attached. The passenger strand can also be cleaved by Ago2 or C3PO endonuclease. The guide strand selection is based on the relative thermodynamic stability of the two strands. The guide strand generally has a relatively unstable 5′ end and has a uridine nucleotide in the first position (98). The loaded miRNA on the RISC (miR-RISC) binds to the complementary sequence of its target messenger RNA (mRNA). Partial complementarity between the miR-RISC and mRNA leads to translational repression, while an exact complementarity leads to mRNA cleavage and degradation (98).

In the non-canonical pathway of miRNA biogenesis, miRNAs can be formed either by the Drosha/DGCR8 independent pathway or the Dicer independent pathway. Mirtrons are an example of the miRNAs formed by the Drosha/DGCR8 independent pathway. The spliceosome cleaves the pri-miRNA of mirtrons, which forms a branched pre-miRNA. Next, the 3′ and 5′ ends of the pre-miRNA are ligated, forming a lariat, which is linearised by a debranching enzyme. After the debranching, the pre-miRNA can be transported to the cytoplasm by the exportin-5-RAN-GTPase complex. Once in the cytoplasm, it is cleaved by Dicer and loaded on Ago to form a mature miRNA (106, 107).

In the Dicer-independent pathway, the microprocessor complex produces the pre-miRNA as short-hairpin RNA transcripts, which are then exported to the cytoplasm. However, the exported transcripts are too short to be processed by Dicer; instead, the entire pre-miRNA duplex is loaded on the Ago complex and undergoes splicing by Ago2 to form the mature miRNA [104] (Figure 2).

### MicroRNAs associated with ageing-induced CVD

**Figure 3:**
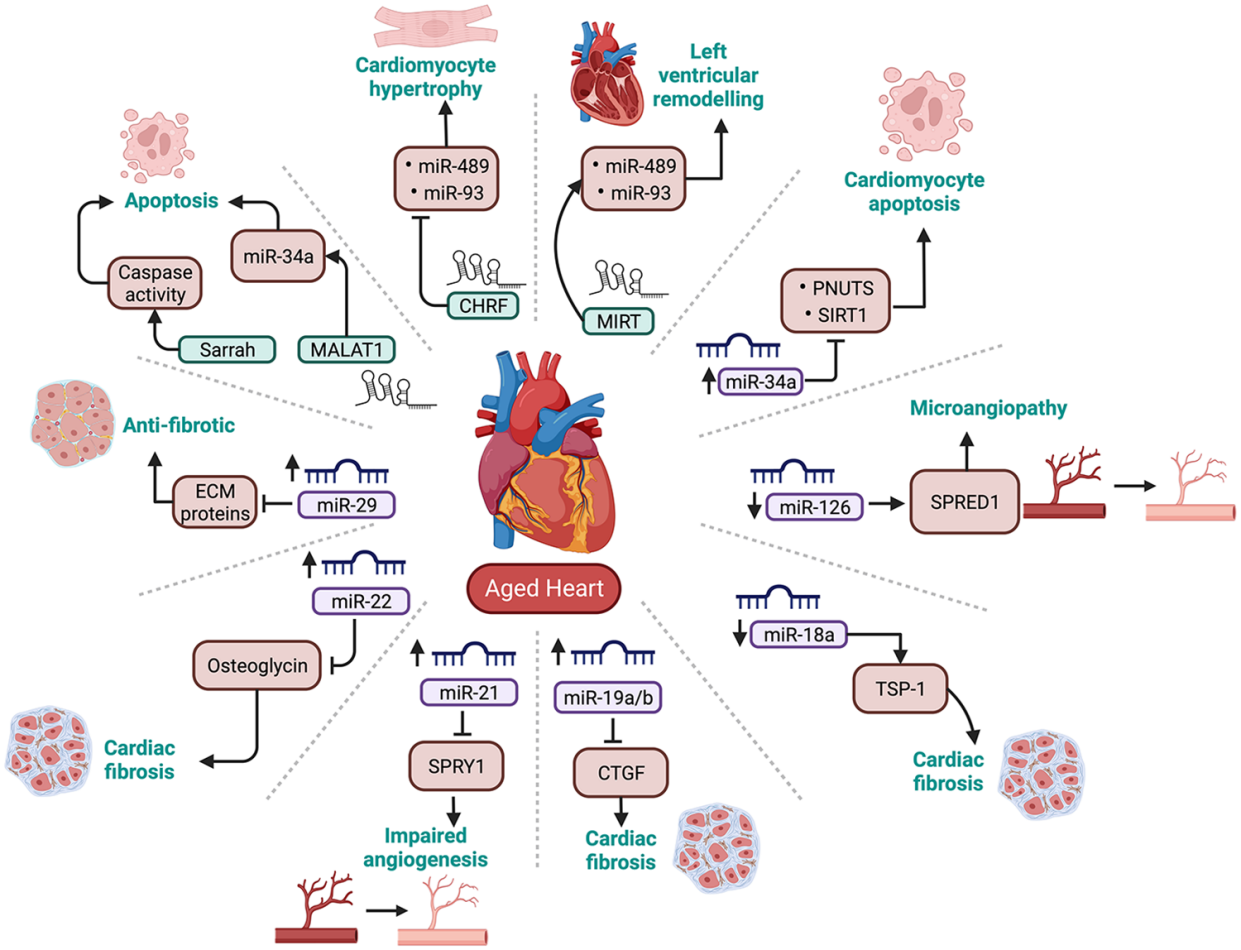
Summary of cardiovascular enriched miRNAs and lncRNAs that are dysregulated with ageing. PNUTS, phosphatase-1 nuclear targeting subunit; SIRT-1, sirtuin 1; TSP-1, thrombospondin-1; CTGF, connective tissue growth factor; SPRY1, sprout protein homolog 1; SPRED1, sprouty related EVH1 domain containing 1; ECM proteins, Extracellular matrix proteins.

While the role of miRNAs in CVD is very well established, it is only recently that researchers have started to explore the role of miRNA dysregulation in the development of age-induced CVD. Therefore, this section will review some key literature on cardiovascular-enriched microRNAs and their role in cardiac ageing (summarised in Figure 3).

#### miR-34a

The miR-34 family consists of 3 miRNAs, miR-34a, -34b and -34c. Among these, miR-34a has been well-characterised and demonstrated to play a significant role in age-related CVD. The gene for miR-34a is located in chromosome 1p36.22 (108). Studies have reported that activation of miR-34a following cardiac injury leads to increased apoptosis of cardiomyocytes (109, 110). Boon et al. observed an upregulation of miR-34a with age in mice cardiomyocytes by inhibiting its direct target phosphatase-1 nuclear-targeting subunit (PNUTS). In support of this, the expression of PNUTS was downregulated in aged mouse hearts (88). PNUTS interacts with telomere repeat factor (TRF2), which protects chromosome ends by enforcing a T-loop structure. In a healthy cell, DNA damage promotes PNUTS translocation to, and repair of, double-stranded DNA breaks during the G2-M checkpoint (111). However, this effect is lost in the aged heart due to the upregulation of miR-34a. Notably, the researchers also demonstrated that forced PNUTS overexpression reduced age- and miR-34a-related markers of DNA damage and protected cardiomyocytes from apoptosis by preventing telomere shortening and promoting the DNA damage response (88).

In addition to apoptosis, miR-34a targets another pro-survival gene, *Sirtuin1* (*SIRT1*) (112). *SIRT1* is a deacetylase enzyme which deacetylates p53, a protein essential for cell cycle and survival. With the repression of SIRT1, p53 is acetylated, leading to an increase in pro-apoptotic proteins like p21 and p53 upregulated modulator of apoptosis (PUMA), eventually leading to apoptosis (113). p53 has a binding site located on the *MiR-34a* gene. Thus, activation of p53 can result in increased transcription of miR-34a, which leads to repression of *SIRT1* and further acetylation of p53. Thus, miR-34a, SIRT1, and p53 have a strong positive feedback loop (113). Interestingly, a recent study from our laboratory showed a marked upregulation of miR-34a in type 2 diabetic hearts from the early stages of the disease (114). Diabetes is a disease which accelerates the ageing of the cells by promoting activation of the pro-senescence signalling cascade (115). Importantly, *in vitro* inhibition of miR-34a markedly reduced high glucose-induced deleterious effects in cardiomyocytes. This evidence suggests that targeting miR-34a could be beneficial for the healthy ageing of the heart.

#### miR-17-92 cluster

The miR-17-92 cluster is transcribed from the miR-17-92 cluster host gene *(MIR17HG)* located in chromosome 13q31.3. The primary transcript is ∼0.8 kb long polycistron which is processed to form seven mature miRNAs, miR-17-3p, -17-5p, -18a, -19a, -19b, -20a and -92a. The miR-17-92 cluster is also referred to as oncomir-1, as its overexpression has been demonstrated to be associated with cancer (116, 117). The miR-17-92 cluster plays a protective role in the heart, with its reduced expression linked to left ventricular hypertrophy, arrhythmias, and heart failure (118, 119). Among all the miRNAs within the cluster, miR-17-3p, -18a, and -19a/b have been shown to have a role in cardiac ageing. The expression of miR-17-3p is downregulated in aged mice hearts, which was associated with the upregulation of its target protein prostate apoptosis response-4 (PAR-4) (120). PAR-4 is a pro-apoptotic protein, and its upregulation negatively affects the expression of anti-apoptotic proteins CCAAT enhancer binding protein beta (CEBPB) and focal adhesion kinase (FAK). Reduced expression of CEBPB and FAK enhances cellular senescence and apoptosis in mouse cardiac fibroblast cells (121, 122).

miR-18a and miR-19a/b are also downregulated in aged mice cardiomyocytes and are associated with an increase in their target proteins, thrombospondin- 1 (TSP-1) and connective tissue growth factor (CTGF), respectively. The increase in TSP-1 and CTGF causes an increase in collagen 1A1 and collagen 3A1 levels leading to the development of cardiac fibrosis (123). This evidence suggests that the members of the miR-17-92 play an essential role in normal cardiac functioning, and their regulation can reduce apoptosis and fibrosis in the aged heart.

#### miR-21

miR-21 is a highly conserved miRNA encoded by the *MIR21* gene, located on chromosome 17q23.2 (124, 125). miR-21 is expressed in cardiac fibroblasts, endothelial cells and vascular smooth muscle cells. Conditions such as cardiac hypertrophy and heart failure upregulate the expression of miR-21. Of note, ageing is associated with both these conditions (126, 127). Therefore, miR-21 upregulation may be associated with cardiac ageing. Sprout protein homolog 1 (SPRY1), a direct target of miR-21, is an inhibitor of the extracellular signal-regulated kinase–mitogen-activated protein (ERK-MAP) kinase. ERK-MAP kinase activates the pathways associated with increased interstitial fibrosis and cardiac hypertrophy (124). Therefore, inhibition of SPRY1 following upregulation of miR-21 may increase fibrosis in the aged heart. Similar to the expression of miR-21 in fibroblasts, the expression of miR-21 is upregulated in the vascular smooth muscles, which inhibits the expression of its other target protein phosphatase and tensin homolog (PTEN). Inhibition of PTEN activates the Phosphatidylinositol-3-kinase and its downstream molecule serine/threonine kinase B (PI3K/AKT) signalling pathway significantly regulates cell proliferation and survival. Activating the PI3K/AKT pathway facilitates the increased proliferation of smooth muscle cells and the increased formation of atherosclerotic plaques (128) Contrary to its role on fibroblasts, miR-21 exhibits pro-angiogenic effects on endothelial cells. Zhang et al. found that the level of miR-21 increased with age in mice, peaking around 18 months, after which it decreased (129). Another study showed that the downregulation of miR-21 was associated with impaired angiogenesis and a reduction in the renewal capacity of endothelial cells (130). Even though several studies have reported the role of miR-21 in CVDs, dysregulation of miR-21 may not be specific to cardiac ageing. This is likely due to the ubiquitous expression of miR-21 in several other tissues. Therefore, dysregulation in the levels of miR-21 cannot be solely associated with cardiac dysfunction (124), especially because it is well-known that miRNAs can transport between cells and in circulation to the distant organ. Thus, a more detailed study is required to ascertain the role of miR-21 in the aged heart (131).

#### miR-22

miR-22 is an oncogenic miRNA, transcribed from the *MIR22* gene located on chromosome 17p13.3 (132). Recently, miR-22 has been identified to have a role in senescence, apoptosis, and angiogenesis (133). To understand the role of miR-22 in cardiac ageing, Jazbutye et al. determined the expression of miR-22 in 4, 24, and 76-week-old mice. They observed an upregulation of miR-22 in fibroblasts with age. Using luciferase assay, they confirmed osteoglycin (mimecan) as the target protein. Interestingly, osteoglycin was downregulated with age (134, 135). Osteoglycin has a protective effect on the heart by reducing collagen production, which eventually reduces fibrosis. This is supported by increased cardiac fibrosis due to the activation of fibroblasts following the downregulation of osteoglycin (134). Like several other miRNAs, studies have demonstrated increased expression of circulating miR-22 in the plasma of patients with acute MI (136) or pancreatic cancer (137). This suggests a possibility for using miR-22 as a potential biomarker for the early diagnosis of ageing-induced cardiac fibrosis.

#### miR-29

miR-29 comprises miR-29a, -29b1, -29b2, -29c, are highly conserved miRNAs. The genes encoding miR-29 family are located on chromosome 7q32.3 (miR-29a and -29b1) and 1q32.2 (miR-29b2 and -29c (138, 139). miR-29 regulates the expression of several proteins such as collagen, fibrillin, and elastin that form the extracellular matrix (ECM) (140). The expression of miR-29 family, especially miR-29a and -29b1 is upregulated with age (140, 141). A study by Heid et al. on aged *Nothobranchius furzeri* hearts observed the upregulation of miR-29a due to increased oxidative stress, a key molecular changes that occur with ageing. Interestingly, upregulation of miR-29a has a cardioprotective effect, as it decreases age-dependent deposition of ECM proteins such as collagens 1A1, 1A2, 11A1, 15A1 and elastin (141, 142). Since increase in collagen deposition is a hallmark sign of cardiac fibrosis, miR-29a acts as anti-fibrotic miRNA (143). While reduced ECM proteins is beneficial against cardiac fibrosis, it has been linked to formation of aneurysm (140, 144). Therefore, upregulation of miR-29 and subsequent reduction in ECM proteins can disrupt the integrity of the vascular wall and can lead to aortic aneurysm (140). Hence a balanced and tissue specific expression levels of miR-29a may be critical. Interestingly, the age-dependent upregulation in the expression of miR-29 and its protective effect was not confined to the heart, but studies have demonstrated these changes in brain as well (145). Although interesting, further studies are required to highlight the functional effects of age-dependent upregulation of miR-29.

The role of miRNAs as regulators of age-associated cardiovascular dysfunction is an exciting area of research. These small noncoding molecules have been linked to several pathologies, such as left ventricular hypertrophy, atherosclerosis, hypertension, MI, and arrhythmia (146).

As discussed above, several studies have established the role of dysregulation of miR-34a, -17-92 cluster, -21, -22, and -29 is a significant factor in the development of age-associated CVDs. However, further investigations are required to accurately determine the role of miRNAs in ageing-induced CVD. For example, it remains unknown whether the miRNAs' dysregulation is cell-specific or occurs in all the tissues. Further, the mechanism and factors that cause dysregulation of these miRNAs with age remain unknown (147). Furthermore, it is vital to determine the role of other well-established cardiovascular enriched and cardiac-specific miRNAs such as miR-1, -208, 133, and -206 in cardiac ageing (148) (summarised in Table 1).

## Long non-coding RNAs (lncRNAs) and ageing

The human genome codes for approximately 16,000 lncRNAs. They are generally more than 200 nucleotides long (149). Unlike miRNAs that repress mRNAs' translation, lncRNAs can upregulate or downregulate gene expression (149). lncRNAs are gaining prominence in cardiovascular studies, with several lncRNAs having been identified and demonstrated to play an essential role in cardiovascular homeostasis. For example, Braveheart (Bvht) is necessary for the differentiation of cardiac stem cells into cardiomyocytes, while FOXF1 adjacent non-coding developmental regulatory RNA (Fendrr) aids in the development of ventricles (150). Similarly, they are also demonstrated to play a role in the development of CVD. lncRNAs such as myosin heavy chain associated RNA transcript (Mhrt), cardiac hypertrophy related factor (Chrf), and HOX antisense intergenic RNA (Hotair) are associated with cardiac hypertrophy. In contrast, cardiac hypertrophy associated with epigenetic regulator (Chaer), maternally expressed gene 3 (Meg3), the non-coding repressor of NFAT (Nron), is associated with heart failure (89). In addition, recent studies have identified a role for Meg3, autophagy promoting factor (Apf), and myocardial infarction-associated transcript (MIRT) in myocardial infarction (89). Despite being extensively studied for their role in several cardiac disorders, the role of lncRNAs in the age-associated development of CVDs is still in its infancy (151). We will next review the biogenesis of lncRNAs and some of the known functions of lncRNAs in association with cardiac ageing.

### lncRNA biogenesis

The biogenesis of lncRNA is different from that of miRNAs but is similar to the formation of mRNAs. LncRNAs are transcribed by RNA polymerase II (89). Most of the lncRNA has a polyadenylated 3′ end and is capped with methyl-guanosine at the 5′ end (152). They undergo alternative splicing and modifications to form lncRNA (153). Based on the cellular fate and function of the specific lncRNA, it can either be localised in the nucleus or transported to the cytoplasm. LncRNAs are exported to the cytoplasm by the nuclear RNA export factor 1 (NXF1) (154). Once in the cytoplasm, according to their function, they are associated with ribosomes located in the mitochondria or exosomes. In general, the biogenesis of lncRNA varies significantly based on the function of the lncRNA (154).

### LncRNAs associated with ageing-induced CVD

#### Metastasis-associated lung adenocarcinoma transcript 1 (MALAT1)

MALAT1 is a highly conserved lncRNA first identified in the metastasis of early-stage non-small cell lung cancer (155). MALAT1 encoded by the *MALAT1* gene located on chromosome 11q.13 (156) maintains basal endothelial cell migration (157), suggesting its crucial role in cardiovascular homeostasis. A recent study by Ruan et. al established a direct link between MALAT1 and miR-34a, where MALAT1 downregulates the expression of miR-34a. As discussed above, miR-34a is upregulated with age which can be attributed to the downregulation of MALAT1 (88, 158). In contrast, Li et al. demonstrated enhanced expression of MALAT1 in cardiac fibroblasts using a rat model of hypertension, which promoted the expression of collagen I and fibronectin leading to myocardial fibrosis (159). Since hypertension is a commonly associated cardiovascular comorbidity with ageing (160) it will be interesting to determine if MALAT1 has differential roles depending on the circumstances and disease conditions.

#### SCOT1-antisense RNA regulated during ageing in the heart (Sarrah)

Sarrah is an anti-apoptotic lncRNA which enhances cardiomyocyte survival and contractility. The expression of Sarrah is downregulated with ageing (161). A downregulation or inhibition of Sarrah activates the caspase activity, thus leading to cardiomyocyte apoptosis (162). *In vivo* overexpression of Sarrah forms a triple helix in the promoter regions and activates transcription of pro-survival genes such as *NRF2* and *GPC6, PDE3A, ITPR2, PARP8, and SSBP2* (161). Besides its role as an anti-apoptotic lncRNA, Sarrah is also associated with angiogenesis, as it stimulates an increase in VEGF signalling (161). To date, there is no direct evidence for the role of Sarrah on cardiac ageing. However, due to its functional role in cardiomyocyte apoptosis, which is increased with cardiac ageing, it is logical that Sarrah will have a role in cardiac ageing, requiring further investigations.

#### Cardiac hypertrophy-related factor (CHRF)

CHRF has been studied to be upregulated in ang-II-induced hypertrophy (163). There are two mechanisms by which CHRF expression causes hypertrophy. The first is by repressing the activity of miR-489, an anti-hypertrophic miRNA (46). CHRF acts as a sponge and inhibits the activity of miR-489, which increases the expression of one of its target proteins, the myeloid differentiation protein (Myd88). Myd88 induces hypertrophy by the NF-*κ*B pathway (46). CHRF also inhibits miR-93, an anti-hypertrophic miRNA. Inhibition of miR-93 increases the expression of Akt3, its target protein, which induces cardiomyocyte hypertrophy (164). The change in the expression of CHRF concerning ageing has not been studied yet, but as discussed above, angII signalling increased with age-induced hypertrophy. Thus, the expression of CHRF might increase with age and can augment hypertrophy in the aged heart (46, 163).

#### Myocardial infarction-associated transcript (MIRT)

MIRT1 and MIRT2 are upregulated in acute myocardial infarction models (163). In addition, MIRT1 and MIRT2 upregulation increased the expression of several genes, such as TGFβ, stat3, and Icam1, all known to be associated with left ventricular remodeling (165). Left ventricular remodeling occurs after MI, in which the LV architecture is altered to distribute pressure overload after MI and an increase in cardiomyocyte hypertrophy (166). Thus, MIRT1 and MIRT2 can play a critical role in LV remodeling after MI, and their regulation can help prevent LV remodeling (165).

LncRNAs and their role in the progression of CVD is relatively new and is less explored than miRNAs (167). LncRNAs have been associated with MI, cardiac hypertrophy, LV remodeling, arrhythmias and other processes (167). Unlike miRNAs, lncRNAs are not highly conserved, and since most of the studies are conducted on mice models or *in vitro*, whether the lncRNAs will have the same function in humans has yet to be fully understood (168). The research on lncRNAs is still in its infancy, and the discovery of new lncRNAs associated with CVDs is rising (summarised in Table 1).

## Future directions, challenges & conclusion

Nc-RNAs such as miRNAs and lncRNAs control gene expression and thus play a critical role in the cell's normal functioning. MiRNAs and lncRNAs are released in the blood, bound in exosomes, microvesicles, or RNA-binding proteins (166). The tissue-specific expression of ncRNAs, dysregulation of their expression in diseases and their release in bodily fluids such as blood and saliva makes them ideal as a potential biomarker for early diagnosis of the disease (169, 170). MiRNAs as therapeutic molecules have been delivered using viral vectors to treat arrhythmia, atherosclerosis, cardiac hypertrophy, fibrosis, and angiogenesis, exhibiting beneficial effects (169). MGN-1374, an antisense oligonucleotide (ASO) therapeutic molecule which inhibits miR-15 and miR-195, which are involved in cardiac ischemic injury and cardiomyopathy, is currently being studied as a potential and novel treatment of acute myocardial infarction (171). MRG-110 is a synthetic molecule being tested to promote angiogenesis by inhibiting anti-angiogenic miR-92a and is currently in phase 1 clinical trials (172). An ASO developed by Haya therapeutics that inhibits the expression of pro-fibrotic lncRNA Wisp2 super-enhancer–associated RNA (Wisper) is also currently in clinical trials (170). LncRNA therapeutics for CVDs are still nascent and restricted to murine models (173). Hence, further studies are required to translate these findings to the clinic.

While our understanding of miRNAs and lncRNAs has been increasing significantly, and several miRNA-based drugs are in clinical trials, we need a better understanding of their mechanisms to identify them as biomarkers for diagnosing CVD. A significant challenge is the varying results in miRNA levels with specific cardiac pathologies (166). For example, some studies indicate the expression of miR-146 increases with age which causes inflammation and oxidative stress, but some studies indicate the expression decreases with age (95). This discrepancy can be attributed to differences in miRNA quantification methods, sample collection and normalisation of sampling parameters, and associated comorbidities such as diabetes and hypertension (174, 175). Different techniques of miRNA quantification, such as qRT-PCR, stem-loop RT-PCR, digital droplet PCR, and RNAseq, can produce different results, as per the method's sensitivity (174). In most studies cited, small nucleolar RNA U6 (snRNA U6) is used as an internal control, but even snRNA U6 may change in some conditions. Hence it is not a reliable internal control (174). Thus, the variability arising from different sampling methods can be reduced by establishing a standardised operating procedure and statistical analysis tools for quantification (166). Other challenges in developing nc-RNA-based therapeutics are RNA instability, difficult delivery of the molecules across the cell membrane, toxicity of exogenous nucleic acids and off-target effects (169, 170). An example of miR-34a can explain the off-target effects of nc-RNA modulation. As discussed above, miR-34a levels increase with age, leading to increased apoptosis of cardiomyocytes. Inhibiting miR-34a to lower apoptosis of cardiomyocytes can be detrimental, as miR-34a is also a tumour suppressor miRNA, and low levels of miR-34a have been associated with carcinomas (176). Inhibiting miR-34a can thus increase the incidence of cancer. Thus, it is essential to consider the function of each nc-RNA in different conditions before modulating its expression. One possibility to overcome this could be to develop a cell-specific delivery strategy. Another way could be the development of transient modulation of the miRNAs rather than a permanent upregulation or knockdown. Despite the challenges, it is beyond doubt that nc-RNAs have the potential as a therapeutic molecule for the treatment of CVDs. Further, combining nc-RNAs based therapeutics with known biomarkers such as cardiac troponins can be more beneficial for diagnosing and treating CVDs (146, 169).

In conclusion, ageing is a dominant risk factor in the progression of CVDs, as even those individuals leading a healthy lifestyle can also develop CVDs as they age. Since the human heart cannot regenerate, it is more susceptible to diseases. Thus, general screening of cardiac functioning tests such as lipid profile and electrocardiogram beginning from a young age can help identify any dysfunction before the disease progresses. Since the dysregulation of miRNAs and lncRNAs occurs with age, tests to screen the levels of miRNAs can be crucial to understanding cardiac functioning. The emergence of miRNAs and lncRNAs in the normal functioning of the cardiovascular system makes them ideal as novel therapeutic targets to prevent the onset of the disease.
